# Distinct expression and function of carotenoid metabolic genes and homoeologs in developing wheat grains

**DOI:** 10.1186/s12870-016-0848-7

**Published:** 2016-07-12

**Authors:** Xiaoqiong Qin, Kathryn Fischer, Shu Yu, Jorge Dubcovsky, Li Tian

**Affiliations:** Department of Plant Sciences, Mail Stop 3, University of California, Davis, CA 95616 USA; Quantitative and Systems Biology Program, University of California, Merced, CA 95343 USA; Howard Hughes Medical Institute, Chevy Chase, MD 20815 USA; Shanghai Key Laboratory of Plant Functional Genomics and Resources, Shanghai Chenshan Botanical Garden, Shanghai, 201602 China; Shanghai Chenshan Plant Science Research Center, Chinese Academy of Sciences, Shanghai, 201602 China

**Keywords:** β-carotene, Carotenoid, Carotenoid cleavage dioxygenase, Endosperm, Grain, Provitamin A, Spatial expression, Wheat

## Abstract

**Background:**

β-carotene, the most active provitamin A molecule produced by plants, plays important roles in human nutrition and health. β-carotene does not usually accumulate in the endosperm (i.e. flour) of mature wheat grains, which is a major food source of calories for humans. Therefore, enriching β-carotene accumulation in wheat grain endosperm will enable a sustainable dietary supplementation of provitamin A. Several metabolic genes affecting β-carotene accumulation have already been isolated from wheat, including phytoene synthase 1 (*PSY1*), lycopene ε-cyclase (*LCYe*) and carotenoid β-ring hydroxylase1/2 (*HYD1/2*).

**Results:**

In this work, we cloned and biochemically characterized two carotenoid cleavage dioxygenases (*CCDs*), *CCD1* and *CCD4*, from wheat. While CCD1 homoeologs cleaved β-apo-8′-carotenal, β-carotene, lutein and zeaxanthin into apocarotenoid products, CCD4 homoeologs were inactive towards these substrates in in vitro assays. When analyzed by real-time qPCR, *PSY1*, *LCYe*, *HYD1*/2 and *CCD1*/*4* homoeologs showed distinct expression patterns in vegetative tissues and sections of developing tetraploid and hexaploid wheat grains, suggesting that carotenoid metabolic genes and homoeologs are differentially regulated at the transcriptional level in wheat.

**Conclusions:**

The CCD1/4 enzyme activity and the spatial-temporal gene expression data provide critical insights into the specific carotenoid metabolic gene homoeologs that control β-carotene accumulation in wheat grain endosperm, thus establishing the knowledge base for generation of wheat varieties with enhanced β-carotene in the endosperm through breeding and genome editing approaches.

**Electronic supplementary material:**

The online version of this article (doi:10.1186/s12870-016-0848-7) contains supplementary material, which is available to authorized users.

## Background

Wheat (*Triticum* spp.), rice and maize constitute the three most widely consumed cereal grains worldwide. The color of wheat grain endosperm (i.e. flour) is determined largely by carotenoid pigments and has been selected according to consumer’s preference during the history of wheat breeding. Tetraploid durum wheat (*T. turgidum*) is used for making pasta and couscous and has been selected for increased concentrations of yellow pigments. Lutein, a non-provitamin A carotenoid exhibiting yellow color, is the major carotenoid molecule present in the endosperm of the mature tetraploid wheat grains. By contrast, hexaploid bread wheat (*T. aestivum*) has been selected for white flour, resulting in low concentrations of total carotenoids in the endosperm of the mature hexaploid wheat grains [1–3]. Therefore, wheat grain endosperm generally lacks provitamin A carotenoids for conversion into vitamin A in mammals.

**Fig. 1:**
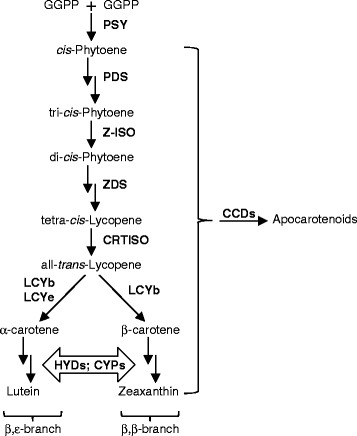
Biosynthetic steps leading to lutein and zeaxanthin production in higher plants. Linear and/or cyclized carotenoids may be converted to apocarotenoids by CCDs. PSY, phytoene synthase; PDS, phytoene desaturase; Z-ISO, ζ-carotene isomerase; ZDS, ζ-carotene desaturase; CRTISO, carotenoid isomerase; LCYb, lycopene β-cyclase; LCYe, lycopene ε-cyclase; HYDs, non-heme di-iron-type carotenoid β-ring hydroxylases; CYPs, cytochrome P450-type carotenoid β- and ε-ring hydroxylases; CCD, carotenoid cleavage dioxygenase

Since humans cannot synthesize vitamin A from basic hydrocarbon building blocks, this essential micronutrient must be obtained from dietary sources, in the form of preformed vitamin A or provitamin A [4–7]. Considering the indispensable role of vitamin A in human nutrition and the importance of wheat flour in supplying dietary calories, enhancing provitamin A accumulation in wheat grain endosperm holds great promise for staple food-based provision/supplementation of vitamin A nutrition. Among the carotenoid molecules that possess provitamin A activities, β-carotene can be converted into vitamin A most efficiently due to its possession of two unmodified β-ionone rings (a structural feature that is essential for vitamin A activity) [5] (Fig. 1). As such, understanding how carotenoid, particularly β-carotene, accumulation is controlled in wheat grain endosperm is crucial for vitamin A biofortification in this tissue.

Several metabolic genes controlling β-carotene accumulation have been isolated from wheat, including *phytoene synthase 1* (*PSY1*) that channels carbon fluxes to carotenoid biosynthesis, *lycopene ε-cyclase* (*LCYe*) that diverts the common biosynthetic precursor lycopene to a competing pathway of lutein biosynthesis, and *β-carotene hydroxylase1/2* (*HYD1/2*) that enable turnover of β-carotene to downstream hydroxylated products [8–10] (Fig. 1).

Besides hydroxylation, β-carotene and its derivatives can also be modified via conversion into smaller molecules (aka. apocarotenoids) by carotenoid cleavage dioxygenases (CCDs) (Fig. 1). Of the four plant CCDs (including CCD1, CCD4, CCD7 and CCD8), coupled actions of CCD7 and CCD8 are required for biosynthesis of strigolactones from 9-*cis*-β-carotene in root tissues [11]. CCD1s identified from different plant species localize to the cytosol and have demonstrated cleavage activities towards all-*trans*-β-carotene (β-carotene hereafter) and additional linear or cyclic (apo)carotenoid substrates at different double bond positions (e.g. C9-C10/C9′-C10′, C7-C8/C7′-C8′ and C5-C6/C5′-C6′) in in vitro enzyme assays [12–32]. Among the CCD1s identified from monocots, rice and maize CCD1s (*Os*CCD1 and *Zm*CCD1) have been functionally characterized [15, 16, 30, 33]. Both enzymes cleave linear (e.g. lycopene) and cyclic (e.g. β-carotene and zeaxanthin) carotenoids symmetrically at C9-C10/C9′-C10′ or C7-C8/C7′-C8′ positions [15, 16, 30]. However, overexpression of *OsCCD1* in Golden Rice endosperm (engineered for high β-carotene accumulation) did not change its β-carotene content significantly, arguing against a role of *Os*CCD1 in carotenoid cleavage in rice endosperm [34]. It remains to be determined whether CCD1 enzymes can transform carotenoids into apocarotenoid products in other cereal grains, such as wheat.

In contrast to the cytosolic localization of CCD1s, CCD4 proteins contain transit peptides for targeting to the plastids [24, 35, 36]. Correlations between reduced *CCD4* gene expression/activity and increased carotenoid accumulation or reduced apocarotenoid volatile emission in floral, fruit or storage tissues have been observed in Arabidopsis, chrysanthemum, grape, peach, potato and saffron, suggesting a role of CCD4s in carotenoid cleavage in these plants [19, 21, 24, 37–40]. However, increased violaxanthin accumulation was detected in RNAi knockdown lines of *StCCD4* (potato *CCD4*), even though *St*CCD4 was unable to cleave violaxanthin in enzyme assays, suggesting that a reduction in CCD4 activity may not lead to the accumulation of its immediate substrates *in planta* [41]. When assayed in vitro, the apple and chrysanthemum CCD4s showed substantial cleavage of β-carotene at C9-C10/C9′-C10′, while the Arabidopsis, rose and osmanthus CCD4s had little conversion of β-carotene into the apocarotenoid products [18, 42]. Although *Cs*CCD4a/b/c of saffron (*Crocus sativus*) all cut β-carotene at C9-C10/C9′-C10′, minor cleavage activities of β-carotene at C7-C8/C7′-C8′ were only observed for *Cs*CCD4c [24, 43]. Lutein, neoxanthin and violaxanthin may also serve as substrates for *Cs*CCD4c as suggested by the carotenoid profiles of *Nicotiana benthamiana* plants transiently overexpressing *CsCCD4c* [43].

Since polyploid wheat has several subgenomes (AA, BB and DD genomes), multiple homoeologs are present for the carotenoid metabolic genes in wheat. While tetraploid wheat (genomes AABB) was formed from the hybridization of *T. urartu* (AA) and an unknown species from the Sitopsis group (BB) about 500,000 years ago, hexaploid wheat (genomes AABBDD) originated from the hybridization of tetraploid wheat (AABB) and diploid wheat (*Aegilops tauschii*) (DD) approximately 8000 to 10,000 years ago [42, 44]. It was proposed that genome duplication and evolution in tetraploid and hexaploid wheat may lead to subfunctionalization or neofunctionalization of gene homoeologs as has been reported for other polyploid plant species [45–47]. However, neofunctionalization occurs less often in polyploid wheat species due to their relatively short evolutionary time spans [42]. Moreover, differences among wheat subgenomes are often manifested as differential expression of homoeologous genes (i.e. different homoeologs of the same gene). Consistent with this notion, we recently observed distinct expression patterns of *HYD1*/*2* and *LCYe* genes and homoeologs in developing tetraploid and hexaploid wheat grains (whole grains were analyzed in this study) [10]. Particularly, *HYD-B1* homoeolog expression strongly resembles those of embryo-specific genes [10]. These data raised the possibility that carotenoid metabolic gene homoeologs could be differentially regulated and may have specific functions in different sections of wheat grains.

The overall objective of this work is to obtain a comprehensive understanding of metabolic genes and homoeologs controlling carotenoid, particularly β-carotene, accumulation in wheat. Since *CCD1/4* had not been isolated and functionally characterized in wheat, we first cloned *CCD1* and *CCD4* homoeologs from wheat and determined the in vitro enzyme activities of their encoded proteins. We then analyzed and compared carotenoid content as well as expression of carotenoid metabolic gene homoeologs (including *PSY1*, *LCYe*, *HYD1*/*2* and *CCD1*/*4*) in vegetative tissues and three sections of developing tetraploid and hexaploid wheat grains.

## Methods

### Plant growth and tissue collection

Seeds of tetraploid wheat var. Kronos and hexaploid wheat breeding line UC1041 were germinated on pre-wetted filter paper and transferred, at 7 d post-germination, to either vermiculite followed by growing in a temperature-controlled growth chamber (for collection of vegetative tissues), or soil followed by growing in a temperature-controlled greenhouse (for collection of grains). Leaf, stem and root tissues of wheat seedlings were harvested after growing in the chamber for three weeks; the collected tissues were immediately frozen in liquid nitrogen. The greenhouse-grown wheat plants were tagged when first anthers became visible from the middle florets of an ear. Developing grains were harvested according to the six defined developmental stages, including watery/1, early milk/2, late milk/3, soft dough/4, hard dough/5 and ripening/6, as previously described [10]. Grains of developmental stages 3–5 were dissected, using forceps and scalpels, into pericarp, endosperm and embryo sections. Grains at stages 1, 2 and 6 are not suitable for dissection as stages 1 and 2 grains are quite small and watery while stage 6 grains are very dry. The dissected grain tissues were immediately frozen in liquid nitrogen. Three biological replicates, each containing pooled sections from multiple grains harvested from several plants, were used for gene expression and metabolite analyses. The frozen vegetative tissues and grain sections were ground into fine powder in liquid nitrogen using mortar and pestle and stored at −80 °C until further analysis.

### RNA extraction and cloning of wheat *CCD1* and *CCD4* homoeologs

Total RNA was extracted from wheat tissues using TRI reagent (Invitrogen, Carlsbad, CA). Quantity and quality of the RNA samples were determined using a Nanodrop® spectrophotometer, according to absorption at 260 nm (RNA quantity) as well as the ratios of A_260_/A_280_ and A_260_/A_230_ (RNA quality). RNA integrity was also evaluated by agarose gel electrophoresis. First strand cDNA synthesis was performed using the BioRad iScript cDNA synthesis kit with mixed random hexamers and oligo(dT)_20_ primers (Hercules, CA).

The coding sequences of wheat *CCD1* or *CCD4* homoeologs are highly similar at the 5′ and 3′ ends. Therefore, the same set of primers, except for *CCD-B1*, was used for amplification of wheat *CCD1* or *CCD4* homoeologs and cloning into the pENTR/D-TOPO vector (Invitrogen). Plasmids extracted from multiple colonies were sequenced in each cloning experiment to identify different homoeologs of *CCD1* or *CCD4*. Upon sequence confirmation, the *CCD1/4* gene homoeologs in pENTR/D-TOPO were then recombined into pDEST17 (Invitrogen) for expression as His-tagged proteins in *E. coli. Arabidopsis thaliana CCD1* (*AtCCD1*) and β-glucuronidase (*GUS*) were also cloned into pDEST17 and used as positive and negative controls for carotenoid cleavage activities, respectively. To improve the solubility of the recombinant CCD4 proteins, His-tagged wheat *CCD4* homoeologs were also subcloned into the pMAL-c2x vector (New England BioLabs, Ipswich, MA) for tagging of the maltose binding protein (MBP). The His-tagged wheat *CCD-A1* was cloned into pMAL-c2x in parallel and used as a control for comparing the cleavage activities between His-tagged and MBP-His-tagged CCD proteins.

When analyzed by TargetP [48], wheat CCD4 homoeologs were predicted to contain N-terminal plastid transit peptides of various lengths (50 aa for CCD-A4, 65 aa for CCD-B4, and 49 aa for CCD-D4). Since it was suggested that removal of subcellular targeting sequences may improve recombinant protein expression in *E. coli* [49, 50], truncated *CCD4* homoeologs (i.e. without the transit peptide-encoding DNA sequences) were also cloned into pENTR/D-TOPO and then pDEST17 for protein expression in *E. coli*. Primers used for cloning wheat *CCD1* and *CCD4* homoeologs are listed in Additional file 1: Table S1.

### Purification of recombinant proteins and CCD enzyme assays

Recombinant plasmids carrying *AtCCD1*, *GUS*, wheat *CCD1* or *CCD4* (full length or truncated) homoeologs were transformed into chemically competent cells of *E. coli* strain Rosetta (DE3)pLysS. The *E. coli* cells were grown at 37 °C until OD_600_ reached 0.6-0.8. Isopropyl β-D-1-thiogalactopyranoside (IPTG) was added to the bacterial cell culture to a final concentration of 0.4 mM. The cells continued to grow at 16 °C for 20 h and were collected by centrifugation at 3000 x g for 20 min. Purification of His-tagged proteins was carried out using Ni-NTA agarose beads (Qiagen, Valencia, CA). MBP-His-tagged proteins were purified using amylose resins (New England BioLabs). Induction and purification of the recombinant proteins were examined by SDS-PAGE.

β-apo-8′-carotenal (Sigma Aldrich, St. Louis, MO), lutein (Chromadex, Irvine, CA) and zeaxanthin (purified from *E. coli* cells expressing pAC-ZEAX) were dissolved in ethanol containing 1.5 % n-octyl-β-D-glucopyranoside (Sigma Aldrich). β-carotene (Sigma Aldrich) was solubilized as previously described with slight modifications [16]. Briefly, β-carotene was dissolved in 20 μl of chloroform with 2 % Tween-80. The mixture was dried under vacuum, resuspended in 20 μl of ethanol, and then added to the reaction mixture. For in vitro CCD enzyme assays, the 200 μl reaction mixture included 20 μg purified recombinant proteins, 100 mM Tris-HCl (pH 7.0), 0.5 mM FeSO_4_, 5 mM ascorbic acid, and the (apo)carotenoid substrate (120 μM β-apo-8′-carotenal, 40 μM β-carotene, 20 μM zeaxanthin or 100 μM lutein). The reaction was allowed to proceed at 30 °C for 2 h and stopped by extraction with ethyl acetate; 20 μl of the ethyl acetate extract was injected on a reverse phase HPLC. The enzyme assays were carried out in triplicate.

Recombinant plasmids carrying wheat *CCD1* or *CCD4* (full length) homoeologs as well as the *GUS* control were also transformed into *E. coli* JM109 (DE3) cells that express pAC-BETA (containing genes for β-carotene production). An overnight culture initiated from a single colony was used to inoculate 25-ml Luria Bertani (LB) with 1 % glucose and appropriate antibiotics. The bacterial culture was grown at room temperature with shaking until OD_600_ reached 0.6. IPTG was added to a final concentration of 0.02 mM and the cells continued to grow at room temperature for an additional 12 h. The growth media were then extracted with ether, dried under N_2_, and resuspended in ethyl acetate; 20 μl of the ethyl acetate resuspension was separated on HPLC. The medium extractions were repeated at least three times from independently transformed and grown bacterial cell cultures.

### HPLC and MS analyses

The HPLC program for analysis of CCD enzyme assay products consisted of three solvents: (A) 20 % acetonitrile, (B) acetonitrile:H_2_O:triethylamine (900:99:1, v/v/v), and (C) ethyl acetate. The gradient elution included: 0–2 min, 100 % A; 2–5 min, 100-50 % A and 0–50 % B; 5–10 min, 50-0 % A and 50–100 % B; 10–13 min, 100-75 % B and 0–25 % C; 13–16 min, 75-30 % B and 25–70 % C; 16–19 min, 30-0 % B and 70–100 % C; 19–21 min, 100 % C; 21–22 min, 0–100 % A and 100-0 % C. Carotenoid extraction from wheat grain tissues as well as HPLC separation and quantification were carried out as previously described [10]. The HPLC program for analysis of bacterial growth media included (A) 50 % acetonitrile, (B) acetonitrile:H_2_O:triethylamine (900:99:1, v/v/v), and (C) ethyl acetate, with the following gradient: 0–10 min, 100-0 % A and 0–100 % B; 10–13 min, 100-75 % B and 0–25 % C; 13–16 min, 75-30 % B and 25–70 % C; 16–19 min, 30-0 % B and 70–100 % C; 19–21 min, 100 % C; 21–22 min, 0–100 % B and 100-0 % C. The flow rate was maintained at 1 ml min^−1^ for all of the HPLC analysis.

Mass spectrometry (MS) analysis was performed on a Thermo Electron LTQ-Orbitrap Hybrid mass spectrometer (Thermo Scientific, Waltham, MA). Product peaks from the CCD enzyme assays were collected from the HPLC runs and used for injection onto the mass spectrometer. An isocratic flow of 50 % (A) H_2_O and 50 % (B) acetonitrile was maintained at 0.2 ml min^−1^ for MS analysis. The mass spectra were acquired by electrospray ionization (ESI) in the positive mode with a mass range of *m/z* 50–1000 Da.

### Phylogenetic analysis

Representative CCD and nine-*cis*-epoxycarotenoid dioxygenase (NCED) proteins from different plant species were selected for multiple sequence alignment using Multiple Sequence Comparison by Log-Expectation (MUSCLE) [51]. The aligned sequences were then used for construction of a neighbor-joining (NJ) tree by MEGA5 with the pairwise deletion method [52]. Robustness of the NJ tree was tested with 1000 rounds of bootstrapping. The rice (*Os*), sorghum (*Sb*) and maize (*Zm*) CCD and NCED sequences were obtained from [33]. The GenBank (non-*A. thaliana* sequences) and AGI (*A. thaliana* sequences) accession numbers are: *At*CCD1, At3g63520; *At*CCD4, At4g19170; *At*CCD7, At2g44990; *At*CCD8, At4g32810; *At*NCED2, At4g18350; *At*NCED3, At3g14440; *At*NCED5, At1g30100; *At*NCED6, At3g24220; *At*NCED9, At1g78390; *Ca*CCD1, DQ157170; *Cc*CCD1, DQ157166; *Cis*CCD1, AB219165; *Cl*CCD1, AB219168; *Cm*CCD1, DQ269467; *Cs*CCD1a, AJ132927; *Cs*CCD1b, EU523661; *Cu*CCD1, AB219164; *Dc*CCD1, DQ192203; *Fa*CCD1, ACA13522; *Of*CCD1, BAJ05401; *Ph*CCD1, AY576003; *Rd*CCD1, ABY47994; *Sl*CCD1A, AY576001; *Sl*CCD1B, AY576002; *Ta*CCD-A1, KU975445; *Ta*CCD-B1, KU975446; *Ta*CCD-D1, KU975447; *Vv*CCD1, AY856353; *Cs*CCD4a, EU523662; *Cs*CCD4b, EU523663; *Cs*CCD4c, JN131499; *Cu*CCD4, BAO18774; *Cxm*CCD4a, ABY60885; *Cxm*CCD4b, BAF36656; *Md*CCD4, ABY47995; *Of*CCD4, ABY60887; *Pp*CCD4, JX309999; *Rd*CCD4, ABY60886; *St*CCD4, XP_006359966; *Ta*CCD-A4, KU975448; *Ta*CCD-B4, KU975449; *Ta*CCD-D4, KU975450; *Bo*LCD, AJ489277; *Cs*ZCD, AJ489276. *At*, *Arabidopsis thaliana*; *Bo*, *Bixa orellana*; *Ca*, *Coffea arabica*; *Cc*, *Coffea canephora; Cis*, *Citrus sinensis*; *Cl*, *Citrus limon*; *Cm*, *Cucumis melo*; *Cs*, *Crocus sativus*; *Cu*, *Citrus unshiu*; *Cxm*, *Chrysanthemum* x *morifolium*; *Dc*, *Daucas carota*; *Fa*, *Fragaria ananassa*; *Md*, *Malus* x *domestica*; *Of*, *Osmanthus fragans*; *Os*, *Oryza sativa*; *Ph*, *Petunia hybrida*; *Pp*, *Prunus persica*; *Rd*, *Rosa* x *damascene*; *Sb*, *Sorghum bicolor*; *Sl*, *Solanum lycopersicum*; *St, Solanum tuberosum*; *Ta*, *Triticum aestivum*; *Vv*, *Vitis vinifera*; *Zm*, *Zea mays*. LCD, Lycopene Cleavage Dioxygenase; ZCD, Zeaxanthin Cleavage Dioxygenase.

### Real-time qPCR analysis

Total RNA was treated with RNase-free DNase I (Fermentas, Glen Burnie, MD). cDNA synthesis was carried out using 5 μg total RNA, random hexamers, and the iScript cDNA synthesis kit (BioRad). Primers specific for wheat *HYD1* and *HYD2* homoeologs as well as the reference genes were reported previously [10]. Primers specific for *PSY1*, *LCYe*, *CCD1* and *CCD4* homoeologs were designed and verified using nullisomic-tetrasomic and ditelosomic lines of hexaploid wheat var. Chinese Spring (Additional file 2: Table S2; Additional file 3: Figure S1). The qPCR products were cloned and sequenced to verify amplification of the target gene homoeologs.

Real-time qPCR analysis was carried out as previously described using the iTaq SYBR® Green Supermix [10]. qPCR amplification efficiency for the target gene homoeologs was in the range of 90 % to 121 %. The relative standard curve method was used for quantification of the transcripts [53]. cDNAs synthesized from whole grains were used for construction of the standard curves in order to allow comparison of homoeologs expressed in different grain sections. Normalization of gene expression was carried out using the geometric mean of two reference genes, Ta2291 and Ta54227, which are stably expressed in different wheat tissues [54].

### Statistical analysis

One way Analysis of Variation (ANOVA) followed by unpaired, two-tailed *t*-test (carotenoid content as well as gene expression in tetraploid wheat) or Tukey’s test (gene expression in hexaploid wheat) were performed. All statistical analysis was carried out using JMP (SAS Institute, Cary, NC).

## Results

### Cloning and biochemical characterization of wheat *CCD1* and *CCD4* homoeologs

**Fig. 2:**
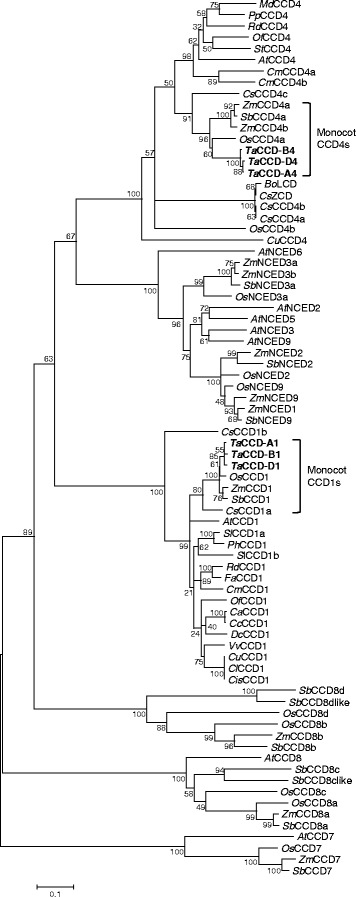
A neighbor-joining tree of wheat CCD1 and CCD4 homoeologs with selected plant carotenoid cleavage enzymes. Bootstrap values are shown next to the branches (1000 replicates). Wheat CCD1 and CCD4 homoeologs are highlighted in bold. CCD, carotenoid cleavage dioxygenase; LCD, lycopene cleavage dioxygenase; NCED, nine-*cis-*epoxycarotenoid dioxygenase; ZCD, zeaxanthin cleavage dioxygenase

Wheat *CCD1* and *CCD4* homoeologs were identified by searching TIGR gene indices as well as the genomic sequences of *Ae. tauschii* and hexaploid wheat var. Chinese Spring using the Arabidopsis *CCD1* and *CCD4* sequences as queries. *CCD1* and *CCD4* homoeologs were assigned to different wheat subgenomes based on the wheat rice synteny and the subgenome assignment was verified using nullisomic-tetrasomic and ditelosomic lines of Chinese Spring (Additional file 3: Figure S1). When compared with CCDs and NCEDs that were previously identified from other plant species, wheat CCD1 and CCD4 homoeologs fell within the respective CCD1 and CCD4 clades with strong support (Fig. 2). Moreover, wheat CCD1 and CCD4 homoeologs clustered closely with their monocot relatives (Fig. 2).

**Fig. 3:**
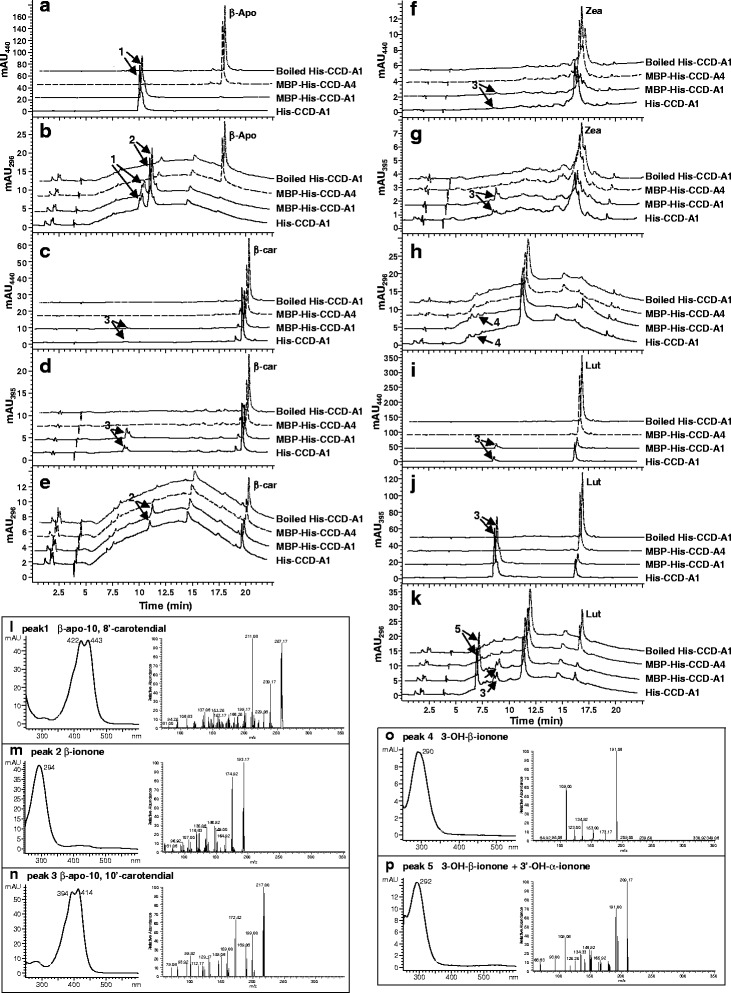
In vitro activities of wheat CCD-A1 and CCD-A4 homoeologs. β-apo-8′-carotenal (β-apo; **a**, **b**), β-carotene (β-car; **c**-**e**), zeaxanthin (Zea; **f**-**h**), and lutein (Lut; **i**-**k**) were used as substrates in the enzyme assays. Product peaks are indicated by arrows. Absorption and mass spectra of the product peaks are shown in (**l**-**p**)

To determine the catalytic activities of wheat CCD1 and CCD4 homoeologs towards (apo)carotenoid substrates, purified recombinant proteins of CCD1/4 were used for in vitro enzyme assays (Additional file 4: Figure S2). The three wheat CCD1 homoeologs cleaved β-apo-8′-carotenal (C_30_ apocarotenoid) at the C9-C10 position to form β-ionone (*m/z* 193.17, [M + H]^+^) and β-apo-10,8′-carotendial (C_17_ dialdehyde; *m/z* 257.17, [M + H]^+^) (Fig. 3; Additional files 5: Figure S3 and Additional file 6: Figure S4). In addition to β-apo-8′-carotenal, wheat CCD1 homoeologs also exhibited significant cleavage activities towards lutein, while relatively small amounts of products were generated when β-carotene and zeaxanthin were provided as substrates (Fig. 3; Additional files 5: Figure S3 and Additional file 6: Figure S4). It should be noted that β-carotene was only partially soluble in the reaction buffer, which may have limited the accessibility of this substrate to the enzymes.

**Fig. 4:**
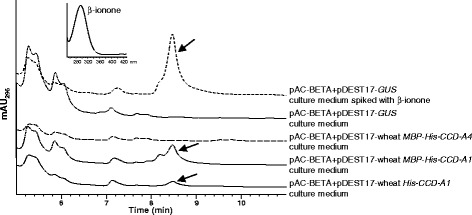
Characterization of wheat CCD-A1 and CCD-A4 activities in β-carotene producing *E. coli* cells. pAC-BETA contains all the genes for β-carotene biosynthesis. Total carotenoids extracted from the growth media of *E. coli* cells co-expressing pAC-BETA and various plasmid constructs were analyzed by HPLC. HPLC elution profiles between 5 and 11 min are shown. Ascending HPLC traces are offset by 12 s for clarity. β-ionone is indicated by arrow. Inset: absorption spectrum of β-ionone

The C_14_ dialdehyde β-apo-10,10′-carotendial (*m/z* 217, [M + H]^+^) was produced in reactions with β-carotene, zeaxanthin or lutein, suggesting symmetrical cleavage of these carotenoids at C9-C10/C9′-C10′ double bonds by wheat CCD1 homoeologs (Fig. 3; Additional files 5: Figure S3 and Additional file 6: Figure S4). Symmetrical cleavage at C9-C10/C9′-C10′ of the carotenoid substrates was also supported by the release of β-ionone (from β-carotene; *m/z* 193.17, [M + H]^+^), 3-OH-β-ionone (from zeaxanthin; major fragment *m/z* 191.08, [M + H]^+^), and 3′-OH-α-ionone + 3-OH-β-ionone (from lutein; *m/z* 209.17, [M + H]^+^) in these reactions (Fig. 3; Additional files 5: Figure S3 and Additional file 6: Figure S4). 3-OH-β-ionone and 3′-OH-α-ionone co-eluted at 6.97 min in the reaction with lutein as substrate, and showed different fragmentation patterns in MS analysis than 3-OH-β-ionone obtained from the zeaxanthin cleavage reaction (Fig. 3) [31, 55]. CCD1 activities towards β-carotene were further verified by analyzing the growth media of *E. coli* cells co-transformed with pAC-BETA (a plasmid that contains β-carotene biosynthetic genes) and CCD1 homoeologs. β-ionone was also found in the growth media of β-carotene producing *E. coli* cells that express wheat CCD1 homoeologs (Fig. 4; Additional file 7: Figure S5).

Unlike the His-tagged CCD1 proteins that partially partitioned to the soluble protein fraction, His-tagged CCD4 proteins were largely insoluble (Additional file 4: Figure S2). CCD4 homoeologs were then expressed as MBP-His-tagged proteins and showed improved protein solubility (Additional file 4: Figure S2). Overall, the purified MBP-His-CCD4 proteins did not exhibit any cleavage activity towards β-apo-8′-carotenal, β-carotene, zeaxanthin and lutein in enzyme assays (Fig. 3; Additional files 5: Figure S3 and Additional file 6: Figure S4). In addition, β-ionone was not detected in growth media of β-carotene producing *E. coli* cells co-expressing wheat CCD4 homoeologs (Fig. 4; Additional file 7: Figure S5). To demine whether the MBP tag may affect protein activities, MBP-His-CCD-A1 was also expressed in *E. coli* and showed improved protein solubility as compared to His-CCD-A1 (Additional file 4: Figure S2). Comparable cleavage activities of the (apo)carotenoid substrates were demonstrated by MBP-His-CCD-A1 and His-CCD-A1, indicating that the MBP tag does not affect the activity of CCD proteins (Figs. 3 and 4). Upon removal of N-terminal plastid transit peptides, solubility of His-truncated CCD-4A/B/D proteins was moderately improved (Additional file 4: Figure S2). However, His-truncated CCD-4A/B/D proteins were not active towards β-apo-8′-carotenal, β-carotene, zeaxanthin and lutein (Additional file 8: Figure S6).

### Carotenoid metabolite profiles in different sections of developing wheat grains

Our previous analysis of whole grains revealed progressively decreased carotenoid accumulation during the 6 defined stages of tetraploid and hexaploid wheat grain development [10]. To further investigate spatial carotenoid accumulation in developing grains, total carotenoids from endosperm, embryo and pericarp sections of tetraploid (var. Kronos) and hexaploid (breeding line UC1041) wheat grains, at stages 3–5 (representing late milk, soft and hard dough stages), were analyzed and compared (Tables 1 and 2).

**Table 1:** Carotenoid composition and content in developing tetraploid wheat (var. Kronos) grain sections

μg carotenoid pigment/g fresh weight of tissue								β,ε/β,β
	Lutein	β-carotene	Zeaxanthin	Neoxanthin	Violaxanthin	Antheraxanthin	Total	
Embryo 3	3.75 ± 0.15^a^	0.36 ± 0.05^a^	0.28 ± 0.04^a^	0.42 ± 0.04^a^	3.06 ± 0.14^a^	2.05 ± 0.17^a^	9.93 ± 0.2^a^	0.61 ± 0.04^a^
Embryo 4	3.52 ± 0.12^a^	0.39 ± 0.06^a^	0.23 ± 0.05^a^	0.38 ± 0.08^a^	2.87 ± 0.22^ab^	1.85 ± 0.08^a^	9.24 ± 0.56^a^	0.62 ± 0.04^a^
Embryo 5	2.46 ± 0.33^b^	0.39 ± 0.02^a^	ND	0.34 ± 0.02^a^	2.55 ± 0.27^b^	1.83 ± 0.02^a^	7.33 ± 0.97^b^	0.51 ± 0.03^b^
Endosperm 3	4.65 ± 0.38^c^	ND	ND	ND	1.51 ± 0.08^c^	ND	6.16 ± 0.4^c^	3.09 ± 0.29^c^
Endosperm 4	4.99 ± 0.64^c^	ND	ND	ND	1.28 ± 0.24^c^	ND	6.27 ± 0.88^bc^	3.92 ± 0.21^c^
Endosperm 5	3.75 ± 0.3^a^	ND	ND	ND	0.48 ± 0.07^d^	ND	4.23 ± 0.25^d^	8.02 ± 1.57^d^
Pericarp 3	18.92 ± 2.62^d^	8.98 ± 1.44^b^	ND	3.72 ± 0.64^b^	7.65 ± 1.02^e^	ND	39.27 ± 5.69^e^	0.93 ± 0.01^e^
Pericarp 4	15.55 ± 1.43^de^	7.78 ± 0.5^b^	ND	2.94 ± 0.3^b^	6.24 ± 0.66^ef^	ND	32.5 ± 2.43^e^	0.92 ± 0.04^e^
Pericarp 5	12.09 ± 1.73^e^	5.01 ± 0.42^c^	ND	2.02 ± 0.15^c^	4.81 ± 0.63^f^	ND	23.93 ± 2.93^f^	1.02 ± 0.04^f^

Data presented are mean ± SD of three biological replicates. Different letters indicate significantly (*P* < 0.05) different carotenoid content or β,ε/β,β ratios in each column. β,ε/β,β, ratio between β,ε- and β,β-branch carotenoids. *ND* not detectable

**Table 2:** Carotenoid composition and content in developing hexaploid wheat (breeding line 1041) grain sections

μg carotenoid pigment/g fresh weight of tissue								β,ε/β,β
	Lutein	β-carotene	Zeaxanthin	Neoxanthin	Violaxanthin	Antheraxanthin	Total	
Embryo 3	2.4 ± 0.33^a^	0.46 ± 0.05^a^	1.03 ± 0.06^a^	0.39 ± 0.02^a^	2.53 ± 0.23^a^	2.32 ± 0.03^a^	9.13 ± 0.69^a^	0.36 ± 0.03^a^
Embryo 4	2.07 ± 0.23^a^	0.37 ± 0.04^a^	1.35 ± 0.05^a^	0.34 ± 0.04^a^	2.42 ± 0.44^a^	3.01 ± 0.04^b^	9.55 ± 0.74^a^	0.28 ± 0.01^b^
Embryo 5	1.83 ± 0.54^a^	0.37 ± 0.05^a^	ND	0.12 ± 0.03^b^	1.84 ± 0.39^a^	2.03 ± 0.27^a^	6.19 ± 1.16^b^	0.41 ± 0.06^a^
Endosperm 3	0.92 ± 0.1^b^	ND	ND	ND	0.9 ± 0.13^b^	ND	1.81 ± 0.23^c^	1.03 ± 0.04^c^
Endosperm 4	0.68 ± 0.02^c^	ND	ND	ND	0.45 ± 0.01^c^	ND	1.13 ± 0.01^d^	1.49 ± 0.07^d^
Endosperm 5	0.5 ± 0.06^d^	ND	ND	ND	0.17 ± 0.01^d^	ND	0.67 ± 0.07^e^	2.96 ± 0.3^e^
Pericarp 3	22.55 ± 3.22^e^	14.34 ± 2.06^b^	ND	4.97 ± 0.84^b^	11.27 ± 1.62^e^	ND	53.13 ± 7.74^f^	0.74 ± 0.01^f^
Pericarp 4	18.07 ± 1.81^e^	10.18 ± 1.01^c^	ND	3.69 ± 0.35^b^	8.84 ± 0.82^f^	ND	40.79 ± 3.99^g^	0.8 ± 0.01^g^
Pericarp 5	8.94 ± 1.67^f^	4.24 ± 0.58^d^	ND	1.15 ± 0.43^c^	3.95 ± 0.4^g^	ND	18.28 ± 3.07^h^	0.95 ± 0.03^c^

Data presented are mean ± SD of three biological replicates. Different letters indicate significantly (*P* < 0.05) different carotenoid content or β,ε/β,β ratios in each column. β,ε/β,β, ratio between β,ε- and β,β-branch carotenoids. *ND* not detectable

The pericarp of wheat grains contain β-carotene, neoxanthin, violaxanthin and lutein, resembling the carotenoid composition of photosynthetic tissues (Tables 1 and 2) [10]. In the pericarp tissue, similar individual and total carotenoid levels were observed between stages 3 and 4 of tetraploid and hexaploid wheat; however, there was a consistent drop in carotenoid content at stage 5, which was significant for β-carotene, neoxanthin and total carotenoids (Tables 1 and 2). On the other hand, an increasing trend in the ratio of β,ε-/β,β-carotene branch (β,ε/β,β) carotenoids was evident for stages 3–5 of grain pericarp (Tables 1 and 2), indicating a shift in carotenoid accumulation favoring the α-carotene/lutein branch relative to the β-carotene/zeaxanthin branch (Fig. 1).

In contrast to pericarp, lutein and violaxanthin were the only detectable carotenoids in the endosperm of tetraploid and hexaploid wheat grains (Tables 1 and 2). The endosperm of tetraploid wheat consistently accumulated higher concentrations of lutein and violaxanthin than that of hexaploid wheat at each grain developmental stage. As grain endosperm matures, violaxanthin decreased more rapidly than lutein and led to a one-fold increase in the β,ε/β,β ratio at stage 5 of tetraploid and hexaploid wheat grains (Tables 1 and 2).

In addition to the carotenoids present in pericarp and endosperm tissues, antheraxanthin and zeaxanthin were also found in embryo (Tables 1 and 2). Particularly, antheraxanthin accounted for about 20 % of total carotenoids in the embryo tissue. As with pericarp and endosperm, total carotenoids in embryo decreased towards the late stage of grain development, contributed mainly by reduced lutein. While the β,ε/β,β ratios in maturing embryo decreased in tetraploid wheat, they remained mostly constant in hexaploid wheat (Tables 1 and 2).

### Expression of carotenoid metabolic gene homoeologs in different sections of developing wheat grains

**Fig. 5:**
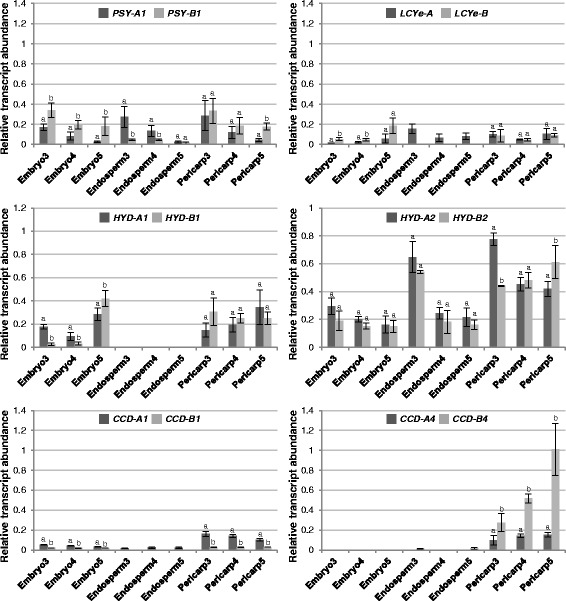
Relative abundance of carotenoid metabolic gene homoeologs in grain sections of tetraploid wheat Kronos. Different letters indicate significant (*P* < 0.05) differences in transcript abundance between two homoeologs of each gene

**Fig. 6:**
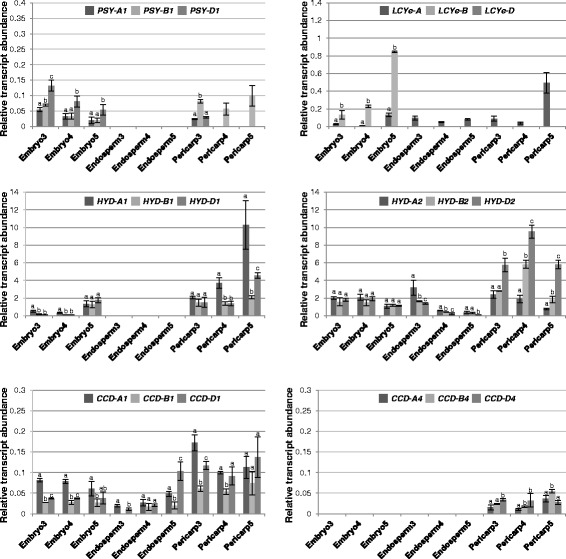
Relative abundance of carotenoid metabolic gene homoeologs in grain sections of hexaploid wheat UC1041. Different letters indicate significant (*P* < 0.05) differences in transcript abundance among three homoeologs of each gene

To assess the spatial contribution of carotenoid metabolic gene homoeologs to grain carotenoid accumulation, expression of *PSY1*, *LCYe*, *HYD1*/*2* and *CCD1*/*4* homoeologs in pericarp, endosperm and embryo tissues of tetraploid and hexaploid wheat grains was analyzed using real-time qPCR (Figs. 5 and 6).

In the pericarp tissue, *PSY-A1* and *PSY-B1* showed comparable and similarly decreased expression during tetraploid wheat grain development, except in stage 5 where the *PSY-B1* transcript level was significantly higher than that of *PSY-A1* (Fig. 5)*.* The preponderance of the B-genome *PSY1* homoeolog was more evident in hexaploid wheat; *PSY-B1* was the major *PSY1* homoeolog expressed in stage 3 and the only *PSY1* transcript detected in stages 4 and 5 (Figs. 5 and 6). While *LCYe*, *HYD1* and *HYD2* homoeologs exhibited similar transcript accumulation through grain development in tetraploid wheat, *HYD1* homoeologs showed increased expression at stage 5 of grain development in hexaploid wheat. In addition, *LCYe-A* was the only *LCYe* homoeolog expressed in the pericarp of hexaploid wheat. Of the *CCD1*/*4* homoeologs, *CCD-B4* expression in pericarp increased gradually towards stage 5 of tetraploid and hexaploid wheat grains. The other *CCD1*/*4* homoeologs showed similar or slightly decreased expression (*CCD-A1*) during tetraploid and hexaploid wheat grain development (Figs. 5 and 6).

In the endosperm tissue, transcripts of all three *PSY1* homoeologs were under the detection limit of real-time qPCR in hexaploid wheat grains, contrasting to the high *PSY1* homoeolog expression found in tetraploid wheat where decreased *PSY-A1* and *PSY-B1* transcript levels were observed in stages 3–5 (Figs. 5 and 6). Interestingly, *LCYe-A* was the only *LCYe* homoeolog expressed in the endosperm of both tetraploid and hexaploid wheat grains. Of the *HYD1/2* genes, *HYD1* homoeolog expression was not detectable, while the *HYD2* homoeologs showed decreased expression in tetraploid and hexaploid wheat (Figs. 5 and 6). Only *CCD-A1* and *CCD-B4* expression was observed in tetraploid wheat grain endosperm. In contrast, all three *CCD1* homoeologs were expressed in the hexaploid wheat grain endosperm, with *CCD-A1* and *CCD-D1* showing increased expression during grain development; on the other hand, none of the *CCD4* homoeologs had detectable transcript accumulation in hexaploid wheat grain endosperm (Figs. 5 and 6).

In the embryo tissue, *PSY-B1* and *PSY-D1* were the most abundant *PSY1* homoeolog in tetraploid and hexaploid wheat, respectively (Figs. 5 and 6). *LCYe-B* in tetraploid wheat as well as *LCYe-A* and *LCYe-B* in hexaploid wheat showed increased expression during grain maturation, particularly at stage 5. While *HYD2* homoeologs had mostly consistent expression in the three grain developmental stages analyzed, enhanced *HYD1* homoeolog expression was observed at the late stage of tetraploid and hexaploid wheat grain development (Figs. 5 and 6). Interestingly, transcripts of *CCD1*, but not *CCD4*, homoeologs were detectable in the embryo tissue of tetraploid and hexaploid wheat grains (Figs. 5 and 6).

### Expression of carotenoid metabolic gene homoeologs in vegetative tissues

**Fig. 7:**
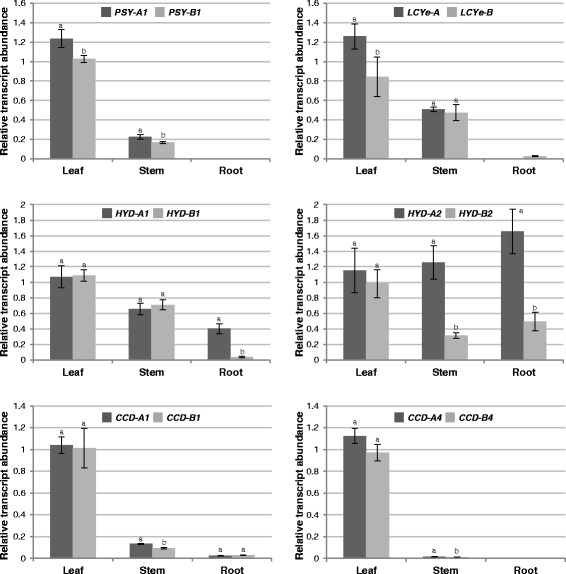
Relative abundance of carotenoid metabolic gene homoeologs in vegetative tissues of tetraploid wheat Kronos. Different letters indicate significant (*P* < 0.05) differences in transcript abundance between two homoeologs of each gene

**Fig. 8:**
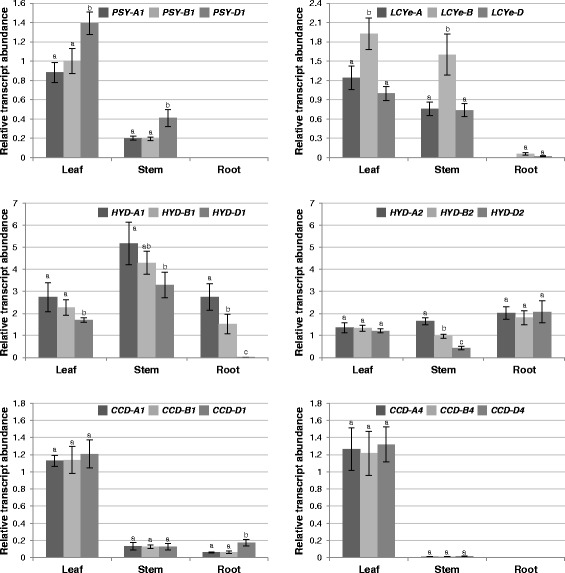
Relative abundance of carotenoid metabolic gene homoeologs in vegetative tissues of hexaploid wheat UC1041. Different letters indicate significant (*P* < 0.05) differences in transcript abundance among three homoeologs of each gene

To determine the genetic factors controlling carotenoid accumulation in vegetative tissues of tetraploid and hexaploid wheat, expression of carotenoid metabolic gene homoeologs in three vegetative tissues, including leaf, stem and root, was analyzed by real-time qPCR (Figs. 7 and 8).

In leaves, *HYD1*/*2* and *CCD1*/*4* homoeologs showed similar expression in both tetraploid and hexaploid wheat. On the other hand, *PSY-A1* and *LCYe*-*A* transcripts were relatively more abundant than their corresponding homoeologs in tetraploid wheat, whereas *PSY-D1* and *LCYe-B* transcripts were relatively more abundant than their corresponding homoeologs in hexaploid wheat (Figs. 7 and 8).

In stems of tetraploid and hexaploid wheat, the relative expression patterns among *PSY1*, *LCYe*, *HYD1* and *CCD1*/*4* homoeologs generally resembled those in leaves. However, expression levels of *PSY1* and *CCD1*/*4* homoeologs were much reduced in stems as compared to leaves (Figs. 7 and 8). Unlike the comparable transcript accumulation of *HYD2* homoeologs in leaves, *HYD-A2* appeared to be the major *HYD2* homoeolog expressed in stems of tetraploid and hexaploid wheat (Figs. 7 and 8).

In roots of tetraploid and hexaploid wheat, *PSY1* and *CCD4* homoeologs were under the limit of detection, and *LCYe* homoeologs were absent or very lowly expressed (Figs. 7 and 8). In tetraploid wheat roots, *HYD-A1* and *HYD-A2* were clearly the major *HYD1* and *HYD2* homoeologs, respectively. In contrast, comparable expression was observed for the three homoeologs of *HYD2*, while *HYD-D1* had only baseline expression in hexaploid wheat roots (Figs. 7 and 8). *CCD-A1* and *CCD-B1* homoeolog expression did not differ significantly for both tetraploid and hexaploid wheat roots, whereas *CCD-D1* showed two-fold higher expression than the other two *CCD1* homoeologs in roots of hexaploid wheat seedlings (Figs. 7 and 8).

## Discussion

Lutein, but not β-carotene, accumulates in the endosperm (flour) of tetraploid and hexaploid wheat grains at varied levels (Tables 1 and 2). While re-directing the carbon flux from lutein to β-carotene formation may be accomplished in wheat grains by blocking the LCYe catalyzed reaction as demonstrated in maize grains and potato tubers [56, 57], it is crucial to understand whether the β-carotene produced could accumulate or will be turned over via hydroxylation by HYDs or cleavage by CCDs (Fig. 1). Our in vitro enzyme assay results showed that wheat CCD1 homoeologs could cleave β-carotene symmetrically, suggesting that CCD1 homoeologs can potentially contribute to the degradation of this provitamin A molecule in grains modified with increased β-carotene production (Figs. 3 and 4; Additional files 5: Figure S3 and Additional file 6: Figure S4). Since lutein can also serve as substrate to CCD1 homoeologs in vitro and it naturally accumulates in wheat grain endosperm, it is possible that the rate of lutein biosynthesis is higher than that of potential degradation by CCD1 homoeologs in this tissue. Alternatively, there could be unknown mechanisms that protect lutein from degradation by CCD1 homoeologs in wheat grain endosperm.

In contrast to CCD1 homoeologs, wheat CCD4 homoeologs did not act on β-carotene, lutein and zeaxanthin (Figs. 3 and 4; Additional files 5: Figure S3, Additional file 6: Figure S4, Additional file 7: Figure S5 and Additional file 8: Figures S6). It remains to be determined whether they could function towards other carotenoid molecules, such as violaxanthin, that were not used for in vitro testing. *At*CCD4 (Arabidopsis CCD4) was previously found in the plastid-localized plastoglobules; in particular, it interacts with a zinc finger protein VAR3 in a protein complex required for normal chloroplast and palisade cell development [35, 58]. It will be interesting to determine whether wheat CCD4 homoeologs are also bound to plastoglobules and cooperates with VAR3-like proteins. Further reverse genetic analysis of wheat CCD4 homoeologs will help elucidate their enzymatic functions *in planta* and the role of their cleavage products in plastid, cell and tissue development.

It was previously shown that maize genotypes with low levels of *CHY* (i.e. *HYD*) and *LCYe* transcripts correlate with high levels of β-carotene accumulation in grains [57, 59], which suggests that carotenoid metabolic gene expression may be indicative of their functions in plants. We therefore examined the expression profiles of carotenoid metabolic gene homoeologs to determine the specific gene homoeologs that control β-carotene accumulation in different sections of developing wheat grains. Overall, the gene expression data indicated that homoeologous carotenoid metabolic genes are regulated at the transcriptional level in sections of wheat grains (Figs. 5, 6, 7 and 8). Additionally, tissue specific expression was more prominent than genome specific expression for the carotenoid metabolic gene homoeologs in wheat (Figs. 5, 6, 7 and 8). Recently, a genome-wide study examined homoeolog expression and genome interplay among three cell types (including endosperm, aleurone layer and transfer cells) of hexaploid wheat grains at 10-, 20- and 30-day after anthesis [60]. Similar to that observed in our gene expression analysis, this study also reported differential expression of homoeologous genes without an overall dominance of a particular subgenome [60]. However, embryo and pericarp tissues were not examined and expression of carotenoid metabolic gene homoeologs were not analyzed in this report [60].

In developing embryo of tetraploid and hexaploid wheat grains, *LCYe-A* and *LCYe-B* homoeologs showed increased expression contrasting to decreased lutein accumulation [10], suggesting that mechanisms other than transcriptional control could also be involved in the regulation of lutein metabolism in wheat grains. Accompanying largely reduced individual and total carotenoids in pericarp at the late stage of wheat grain development, *CCD-B4* expression increased sharply, raising the possibility that it may be responsible for carotenoid degradation in pericarp during grain dehydration. This observation is consistent with a recent study in Arabidopsis where *AtCCD4* expression rose during seed drying (comparable to wheat grain stages 4–5) and was shown genetically as a major contributor of β-carotene degradation during Arabidopsis seed desiccation [21]. However, since whole Arabidopsis seeds were used in this study, the spatial location of *AtCCD4* expression and β-carotene accumulation within Arabidopsis seeds were not determined.

Our gene expression results also provided important insights for future attempts to increase β-carotene content in wheat grain endosperm. In the endosperm of tetraploid and hexaploid wheat grains, *HYD1* homoeologs were not expressed; *CCD1* and *CCD4* homoeologs were either non-detectable or had low level expression, thus may not impact carotenoid accumulation in this tissue. On the other hand, there were significant transcript accumulation of *LCYe-A* (but not *LCYe-B* or *LCYe-D*) and *HYD2* homoeologs, suggesting that downregulation or loss-of-function of these homoeologs may be sufficient to result in more β-carotene in the endosperm of tetraploid and hexaploid wheat grains. Since the total carotenoid content in hexaploid wheat grain endosperm is low, more carbons will also need to the directed to the carotenoid biosynthetic pathway, such as introducing *PSY1* genes with higher expression or activity, to increase β-carotene levels in hexaploid wheat grain endosperm.

Considering the critical roles that carotenoids play in light harvesting and photoprotection, it is important to ensure that downregulation of specific carotenoid metabolic gene homoeologs in grain endosperm will not compromise carotenoid biosynthesis and function in the photosynthetic tissue. *LCYe*, *HYD1/2* and *CCD1*/*4* homoeologs showed comparable expression in leaves (Figs. 7 and 8), suggesting that loss/reduction of activities of specific carotenoid metabolic gene homoeologs in the photosynthetic tissue could be compensated for by the overlapping activities of homologous (e.g. *HYD1* and *HYD2*) and/or homoeologous (e.g. different homoeologs of *HYD2*) genes.

## Conclusion

Taken together, the CCD1/4 enzyme activity and the spatial gene expression analyses suggested that reduced expression of *LCYe-A* and one or more of the loci, including *HYD-A2*, *HYD-B2*, and *CCD1* homoeologs (only for hexaploid wheat), could lead to β-carotene enrichment in the endosperm of wheat grains without compromising carotenoid metabolism in leaves. Identification of the specific carotenoid metabolic gene homoeologs controlling β-carotene accumulation will facilitate efficient and effective provitamin A biofortification of wheat grains through plant breeding and genome editing technologies.

## Abbreviations

ANOVA, analysis of variation; CCD, carotenoid cleavage dioxygenase; ESI, electrospray ionization; GUS, β-glucuronidase; HYD, carotenoid β-ring hydroxylase; IPTG, isopropyl β-D-1-thiogalactopyranoside; LCD, lycopene cleavage dioxygenase; LCYe, lycopene ε-cyclase; MBP, maltose binding protein; MS, mass spectrometry; MUSCLE, multiple sequence comparison by log-expectation; NCED, nine-*cis*-epoxycarotenoid dioxygenase; NJ, neighbor-joining; PSY, phytoene synthase; ZCD, zeaxanthin cleavage dioxygenase

## Additional files

Additional file 1: Table S1. Primers used for cloning of wheat *CCD1* and *CCD4* homoeologs. (DOCX 15 kb) Additional file 2: Table S2. Homoeolog-specific primers used for real-time qPCR analysis. (PDF 17 kb) Additional file 3: Figure S1. Verification of homoeolog-specific primers using tetrasomic- nullisomic and ditelosomic lines of hexaploid wheat var. Chinese Spring. (PDF 377 kb) Additional file 4: Figure S2.
*E. coli* expression and purification of wheat CCD1 and CCD4 proteins. (PDF 187 kb) Additional file 5: Figure S3. In vitro activities of wheat CCD-B1 and CCD-B4 homoeologs. (PDF 148 kb) Additional file 6: Figure S4. In vitro activities of wheat CCD-D1 and CCD-D4 homoeologs. (PDF 148 kb) Additional file 7: Figure S5. Characterization of wheat CCD-B1, CCD-B4, CCD-D1 and CCD-D4 activities in β-carotene producing *E. coli* cells. (PDF 45 kb) Additional file 8: Figure S6. In vitro activities of truncated wheat CCD4 homoeologs. (PDF 259 kb)

## References

[1] Panfili G, Fratianni A, Irano M (2004). Improved normal-phase high-performance liquid chromatography procedure for the determination of carotenoids in cereals. J Agric Food Chem.

[2] Hentschel V, Kranl K, Hollmann J, Lindhauer M, Bohm V, Bitsch R (2002). Spectrophotometric determination of yellow pigment content and evaluation of carotenoids by high-performance liquid chromatography in durum wheat grain. J Agric Food Chem.

[3] Calucci L, Capocchi A, Galleschi L, Ghiringhelli S, Pinzino C, Saviozzi F, Zandomeneghi M (2004). Antioxidants, free radicals, storage proteins, puroindolines, and proteolytic activities in bread wheat (*Triticum aestivum*) seeds during accelerated aging. J Agric Food Chem.

[4] Britton G, Britton G, Liaaen-Jensen S, Pfander H (2008). Functions of intact carotenoids. Carotenoids Volume 4: Natural Functions.

[5] Britton G, Britton G, Liaaen-Jensen S, Pfander H (2009). Vitamin A and vitamin A deficiency. Carotenoids Volume 5: Nutrition and Health.

[6] Bendich A (2004). From 1989 to 2001: what have we learned about the “biological actions of beta-carotene”?. J Nutr.

[7] Mora JR, Iwata M, von Andrian UH (2008). Vitamin effects on the immune system: vitamins A and D take centre stage. Nat Rev Immunol.

[8] Howitt C, Cavanagh C, Bowerman A, Cazzonelli C, Rampling L, Mimica J, Pogson B (2009). Alternative splicing, activation of cryptic exons and amino acid substitutions in carotenoid biosynthetic genes are associated with lutein accumulation in wheat endosperm. Funct Integr Genomics.

[9] Zhang W, Dubcovsky J (2008). Association between allelic variation at the *Phytoene synthase 1* gene and yellow pigment content in the wheat grain. Theor Appl Genet.

[10] Qin X, Zhang W, Dubcovsky J, Tian L (2012). Cloning and comparative analysis of carotenoid β-hydroxylase genes provides new insights into carotenoid metabolism in tetraploid (*Triticum turgidum* ssp. *durum*) and hexaploid (*Triticum aestivum*) wheat grains. Plant Mol Biol.

[11] Alder A, Jamil M, Marzorati M, Bruno M, Vermathen M, Bigler P, Ghisla S, Bouwmeester H, Beyer P, Al-Babili S (2012). The path from β-carotene to carlactone, a strigolactone-like plant hormone. Science.

[12] Auldridge ME, Block A, Vogel JT, Dabney-Smith C, Mila I, Bouzayen M, Magallanes-Lundback M, DellaPenna D, McCarty DR, Klee HJ (2006). Characterization of three members of the Arabidopsis carotenoid cleavage dioxygenase family demonstrates the divergent roles of this multifunctional enzyme family. Plant J.

[13] Auldridge ME, McCarty DR, Klee HJ (2006). Plant carotenoid cleavage oxygenases and their apocarotenoid products. Curr Opin Plant Biol.

[14] Mathieu S, Terrier N, Procureur J, Bigey F, Günata Z (2005). A carotenoid cleavage doxygenase from *Vitis vinifera* L.: functional characterization and expression during grape berry development in relation to C_13_-norisoprenoid accumulation. J Exp Bot.

[15] Vogel J, Tan B, McCarty D, Klee H (2008). The carotenoid cleavage dioxygenase 1 enzyme has broad substrate specificity, cleaving multiple carotenoids at two different bond positions. J Biol Chem.

[16] Ilg A, Beyer P, Al-Babili S (2009). Characterization of the rice carotenoid cleavage dioxygenase 1 reveals a novel route for geranial biosynthesis. FEBS J.

[17] Gang D (2005). Evolution of flavors and scents. Annu Rev Plant Biol.

[18] Huang F, Molnár P, Schwab W (2009). Cloning and functional characterization of carotenoid cleavage dioxygenase 4 genes. J Exp Bot.

[19] Ohmiya A, Kishimoto S, Aida R, Yoshioka S, Sumitomo K (2006). Carotenoid Cleavage Dioxygenase (CmCCD4a) contributes to white color formation in Chrysanthemum petals. Plant Physiol.

[20] Ohmiya A (2009). Carotenoid cleavage dioxygenases and their apocarotenoid products in plants. Plant Biotechnol.

[21] Gonzalez-Jorge S, Ha S, Magallanes-Lundback M, Gilliland L, Zhou A, Lipka A, Nguyen Y, Angelovici R, Lin H, Cepela J (2013). Carotenoid cleavage dioxygenase4 is a negative regulator of β-carotene content in Arabidopsis seeds. Plant Cell.

[22] Rodrigo M, Alquézar B, Alós E, Medina V, Carmona L, Bruno M, Al-Babili S, Zacarías L (2013). A novel carotenoid cleavage activity involved in the biosynthesis of citrus fruit-specific apocarotenoid pigments. J Exp Bot.

[23] Bouvier F, Suire C, Mutterer J, Camara B (2003). Oxidative remodeling of chromoplast carotenoids: identification of the carotenoid dioxygenase *CsCCD* and *CsZCD* genes involved in crocus secondary metabolite biogenesis. Plant Cell.

[24] Rubio A, Rambla JL, Santaella M, Gómez MD, Orzaez D, Granell A, Gómez-Gómez L (2008). Cytosolic and plastoglobule-targeted carotenoid dioxygenases from *Crocus sativus* are both involved in β-ionone release. J Biol Chem.

[25] Simkin AJ, Underwood BA, Auldridge M, Loucas HM, Shibuya K, Schmelz E, Clark DG, Klee HJ (2004). Circadian regulation of the PhCCD1 carotenoid cleavage dioxygenase controls emission of β-ionone, a fragrance volatile of petunia flowers. Plant Physiol.

[26] Simkin AJ, Schwartz SH, Auldridge M, Taylor M, Klee HJ (2004). The tomato *carotenoid cleavage dioxygenase 1* genes contribute to the formation of the flavor volatiles β-ionone, pseudoionone, and geranylacetone. Plant J.

[27] Kato M, Matsumoto H, Ikoma Y, Okuda H, Yano M (2006). The role of carotenoid cleavage dioxygenases in the regulation of carotenoid profiles during maturation in citrus fruit. J Exp Bot.

[28] Ibdah M, Azulay Y, Portnoy V, Wasserman B, Bar E, Meir A, Burger Y, Hirschberg J, Schaffer A, Katzir N (2006). Functional characterization of *CmCCD1*, a carotenoid cleavage dioxygenase from melon. Phytochemistry.

[29] Simkin AJ, Moreau H, Kuntz M, Pagny G, Lin C, Tanksley S, McCarthy J (2008). An investigation of carotenoid biosynthesis in *Coffea canephora* and *Coffea arabica*. J Plant Physiol.

[30] Sun Z, Hans J, Walter M, Matusova R, Beekwilder J, Verstappen F, Ming Z, van Echtelt E, Strack D, Bisseling T (2008). Cloning and characterisation of a maize carotenoid cleavage dioxygenase (ZmCCD1) and its involvement in the biosynthesis of apocarotenoids with various roles in mutualistic and parasitic interactions. Planta.

[31] García-Limones C, Schnäbele K, Blanco-Portales R, Luz Bellido M, Caballero JL, Schwab W, Muñoz-Blanco J (2008). Functional characterization of FaCCD1: a carotenoid cleavage dioxygenase from strawberry involved in lutein degradation during fruit ripening. J Agric Food Chem.

[32] Baldermann S, Kato M, Kurosawa M, Kurobayashi Y, Fujita A, Fleischmann P, Watanabe N (2010). Functional characterization of a carotenoid cleavage dioxygenase 1 and its relation to the carotenoid accumulation and volatile emission during the floral development of *Osmanthus fragrans* Lour. J Exp Bot.

[33] Vallabhaneni R, Bradbury LMT, Wurtzel ET (2010). The carotenoid dioxygenase gene family in maize, sorghum, and rice. Arch Biochem Biophys.

[34] Ilg A, Yu Q, Schaub P, Beyer P, Al-Babili S (2010). Overexpression of the rice *carotenoid cleavage dioxygenase 1* gene in Golden Rice endosperm suggests apocarotenoids as substrates *in planta*. Planta.

[35] Ytterberg AJ, Peltier J-B, van Wijk KJ (2006). Protein profiling of plastoglobules in chloroplasts and chromoplasts. A surprising site for differential accumulation of metabolic enzymes. Plant Physiol.

[36] Ma G, Zhang L, Matsuta A, Matsutani K, Yamawaki K, Yahata M, Wahyudi A, Motohashi R, Kato M (2013). Enzymatic formation of β-citraurin from β-cryptoxanthin and zeaxanthin by Carotenoid Cleavage Dioxygenase4 in the flavedo of citrus fruit. Plant Physiol.

[37] Campbell R, Ducreux LJM, Morris WL, Morris JA, Suttle JC, Ramsay G, Bryan GJ, Hedley PE, Taylor MA (2010). The metabolic and developmental roles of carotenoid cleavage dioxygenase4 from potato. Plant Physiol.

[38] Brandi F, Bar E, Mourgues F, Horvath G, Turcsi E, Giuliano G, Liverani A, Tartarini S, Lewinsohn E, Rosati C (2011). Study of ‘Redhaven’ peach and its white-fleshed mutant suggests a key role of CCD4 carotenoid dioxygenase in carotenoid and norisoprenoid volatile metabolism. BMC Plant Biol.

[39] Falchi R, Vendramin E, Zanon L, Scalabrin S, Cipriani G, Verde I, Vizzotto G, Morgante M (2013). Three distinct mutational mechanisms acting on a single gene underpin the origin of yellow flesh in peach. Plant J.

[40] Lashbrooke JG, Young PR, Dockrall SJ, Vasanth K, Vivier MA (2013). Functional characterisation of three members of the Vitis vinifera L. carotenoid cleavage dioxygenase gene family. BMC Plant Biol.

[41] Bruno M, Beyer P, Al-Babili S (2015). The potato carotenoid cleavage dioxygenase 4 catalyzes a single cleavage of β-ionone ring-containing carotenes and non-epoxidated xanthophylls. Arch Biochem Biophys.

[42] Dubcovsky J, Dvorak J (2007). Genome plasticity a key factor in the success of polyploid wheat under domestication. Science.

[43] Rubio-Moraga A, Rambla J, Fernández-de-Carmen A, Trapero-Mozos A, Ahrazem O, Orzáez D, Granell A, Gómez-Gómez L (2014). New target carotenoids for CCD4 enzymes are revealed with the characterization of a novel stress-induced carotenoid cleavage dioxygenase gene from *Crocus sativus*. Plant Mol Biol.

[44] Huang S, Sirikhachornkit A, Su X, Faris J, Gill B, Haselkorn R, Gornicki P (2002). Genes encoding plastid acetyl-CoA carboxylase and 3-phosphoglycerate kinase of the Triticum/Aegilops complex and the evolutionary history of polyploid wheat. Proc Natl Acad Sci U S A.

[45] Akhunova A, Matniyazov R, Liang H, Akhunov E (2010). Homoeolog-specific transcriptional bias in allopolyploid wheat. BMC Genomics.

[46] Nomura T, Ishihara A, Yanagita R, Endo T, Iwamura H (2005). Three genomes differentially contribute to the biosynthesis of benzoxazinones in hexaploid wheat. Proc Natl Acad Sci U S A.

[47] Bottley A, Koebner RMD (2008). Variation for homoeologous gene silencing in hexaploid wheat. Plant J.

[48] Emanuelsson O, Brunak S, von Heijne G, Nielsen H (2007). Locating proteins in the cell using TargetP, SignalP and related tools. Nat Protoc.

[49] Koch B, Sibbesen O, Halkier BA, Svendsen I, Lindberg Møller B (1995). The primary sequence of cytochrome P450tyr, the multifunctional *N*-hydroxylase catalyzing the conversion of L-tyrosine to *p*-hydroxyphenylacetaldehyde oxime in the biosynthesis of the cyanogenic glucoside dhurrin in *Sorghum bicolor* (L.) Moench. Arch Biochem Biophys.

[50] Hull AK, Celenza JL (2000). Bacterial epression and purification of the Arabidopsis NADPH–cytochrome P450 reductase ATR2. Protein Express Purif.

[51] Edgar R (2004). MUSCLE: multiple sequence alignment with high accuracy and high throughput. Nucl Acids Res.

[52] Tamura K, Peterson D, Peterson N, Stecher G, Nei M, Kumar S (2011). MEGA5: molecular evolutionary genetics analysis using maximum likelihood, evolutionary distance, and maximum parsimony methods. Mol Biol Evol.

[53] Applied Biosystems (2008). Guide to performing relative quantification of gene expression using real-time quantitative PCR.

[54] Paolacci A, Tanzarella O, Porceddu E, Ciaffi M (2009). Identification and validation of reference genes for quantitative RT-PCR normalization in wheat. BMC Mol Biol.

[55] Mein JR, Dolnikowski GG, Ernst H, Russell RM, Wang X-D (2011). Enzymatic formation of apo-carotenoids from the xanthophyll carotenoids lutein, zeaxanthin and β-cryptoxanthin by ferret carotene-9′,10′-monooxygenase. Arch Biochem Biophys.

[56] Diretto G, Tavazza R, Welsch R, Pizzichini D, Mourgues F, Papacchioli V, Beyer P, Giuliano G (2006). Metabolic engineering of potato tuber carotenoids through tuber-specific silencing of lycopene epsilon cyclase. BMC Plant Biol.

[57] Harjes C, Rocheford T, Bai L, Brutnell T, Kandianis C, Sowinski S, Stapleton A, Vallabhaneni R, Williams M, Wurtzel E (2008). Natural genetic variation in lycopene epsilon cyclase tapped for maize biofortification. Science.

[58] Næsted H, Holm A, Jenkins T, Nielsen HB, Harris CA, Beale MH, Andersen M, Mant A, Scheller H, Camara B (2004). Arabidopsis VARIEGATED 3 encodes a chloroplast-targeted, zinc-finger protein required for chloroplast and palisade cell development. J Cell Sci.

[59] Yan J, Kandianis C, Harjes C, Bai L, Kim E, Yang X, Skinner D, Fu Z, Mitchell S, Li Q (2010). Rare genetic variation at *Zea mays crtRB1* increases beta-carotene in maize grain. Nat Genet.

[60] Pfeifer M, Kugler KG, Sandve SR, Zhan B, Rudi H, Hvidsten TR, Consortium IWGS, Mayer KFX, Olsen O-A (2014). Genome interplay in the grain transcriptome of hexaploid bread wheat. Science.

