# Supplementary material for: Distinct expression and function of carotenoid metabolic genes and homoeologs in developing wheat grains
Source: BMC Plant Biol. 2016 Jul 12;16:155. doi: 10.1186/s12870-016-0848-7 (PMC4943016; doi:10.1186/s12870-016-0848-7)
Supplement: Additional file 1: Table S1. — Primers used for cloning of wheat CCD1 and CCD4 homoeologs. (DOCX 15 kb) [file 12870_2016_848_MOESM1_ESM.docx]

**Table S1.** Primers used for cloning of wheat *CCD1* and *CCD4* homeologs.

| Homeolog | Forward (5’🡪3’) | Reverse (5’🡪3’) |
| --- | --- | --- |
| *CCD-A1* | CACCATGGGGGAAGCGGTGGCC | TCACACCTCTGCTTGCTGATGTAC |
| *CCD-B1* | CACCATGGGAGAAGCGGTGGCG | TCACACCTCTGCTTGCTGATGTAC |
| *CCD-D1* | CACCATGGGGGAAGCGGTGGCC | TCACACCTCTGCTTGCTGATGTAC |
| *CCD-A4* (full length) | CACCATGTCTGATCTCTACCCCGC | TAGCAATTCTGCAGGCTGGA |
| *CCD-A4* (without transit peptide) | CACCGCCGCCACCGCCACCG | TAGCAATTCTGCAGGCTGGA |
| *CCD-B4* (full length) | CACCATGTCTGATCTCTACCCCGC | TAGCAATTCTGCAGGCTGGA |
| *CCD-B4* (without transit peptide) | CACCCAACAAGAAACGGAAG | TAGCAATTCTGCAGGCTGGA |
| *CCD-D4* (full length) | CACCATGTCTGATCTCTACCCCGC | TAGCAATTCTGCAGGCTGGA |
| *CCD-D4* (without transit peptide) | CACCGCTGCCGCCACCGCCA | TAGCAATTCTGCAGGCTGGA |
