# Supplementary material for: Distinct expression and function of carotenoid metabolic genes and homoeologs in developing wheat grains
Source: BMC Plant Biol. 2016 Jul 12;16:155. doi: 10.1186/s12870-016-0848-7 (PMC4943016; doi:10.1186/s12870-016-0848-7)
Supplement: Additional file 2: Table S2. — Homoeolog-specific primers used for real-time qPCR analysis. (PDF 17 kb) [file 12870_2016_848_MOESM2_ESM.pdf]

Table S2. Homeolog-specific primers used for real-time qPCR analysis.

Homeolog	Forward (5’→3’)	Reverse (5’→3’)	Amplicon (bp)
PSY-A1	GAATGAAACATGGCAGTGTG	AGAACTTCATGTCATGCCTA	120
PSY-B1	GATGAAGATGGTCAGGTTAG	CAGAACTTCATGTTATGCATG	161
PSY-D1	AAAGAAACAGGGCGGTGTC	AGCCCTTCGTGTCATGGATT	111
LCYe-A	GTTCGACGCTTTCGTCGGT	TCTCGCAGGCTAGTCAAATG	175
LCYe-B	GGATATCACCTGCCATGTG	CACGCTCCCACTTTTGGCA	144
LCYe-D	GGATATCACCTGCCATGTG	AACCTAAAATTAAACCATGGCA	102
CCD-A1	TGTCAACGAGGAACAACTGG	GCAATGCTACTAATTTAACGC	85
CCD-B1	AGAAGCAGCTTGAAGTCGGT	GATAGCCGTCATCTTCTTGA	126
CCD-D1	TGTCAACGAGGAACAACTGG	TCTGCAGCATGCAGACCAC	98
CCD-A4	CCGAGCTCCGGTCACAGC	ATACATCGCGCGATCAATCC	89
CCD-B4	TCCACGGCCTATTCGTCACT	CTATTATACATCGTGGCGCG	121
CCD-D4	CCGAGCTCCGGTCACAGC	TCTACTATTGTACATTGTCGCA	103
