# Supplementary material for: Distinct expression and function of carotenoid metabolic genes and homoeologs in developing wheat grains
Source: BMC Plant Biol. 2016 Jul 12;16:155. doi: 10.1186/s12870-016-0848-7 (PMC4943016; doi:10.1186/s12870-016-0848-7)
Supplement: Additional file 3: Figure S1. — Verification of homoeolog-specific primers using tetrasomic- nullisomic and ditelosomic lines of hexaploid wheat var. Chinese Spring. (PDF 377 kb) [file 12870_2016_848_MOESM3_ESM.pdf]

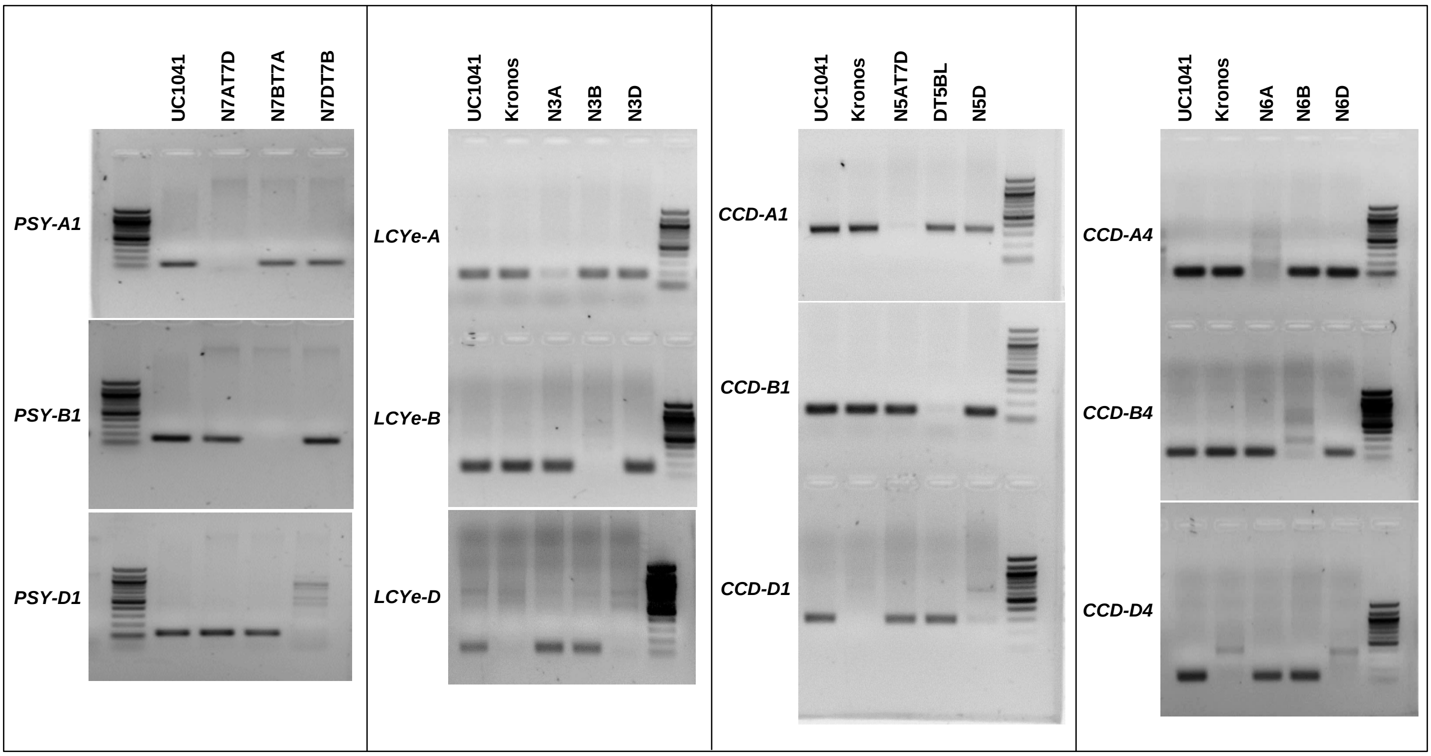

Figure S1. Verification of homeolog-specific primers using tetrasomic-nullisomic and ditelosomic lines of hexaploid wheat var. Chinese Spring. PCR amplifications using genomic DNA extracted from hexaploid wheat breeding line UC1041 and tetraploid wheat var. Kronos are also shown.
