# Supplementary material for: Distinct expression and function of carotenoid metabolic genes and homoeologs in developing wheat grains
Source: BMC Plant Biol. 2016 Jul 12;16:155. doi: 10.1186/s12870-016-0848-7 (PMC4943016; doi:10.1186/s12870-016-0848-7)
Supplement: Additional file 4: Figure S2. — E. coli expression and purification of wheat CCD1 and CCD4 proteins. (PDF 187 kb) [file 12870_2016_848_MOESM4_ESM.pdf]

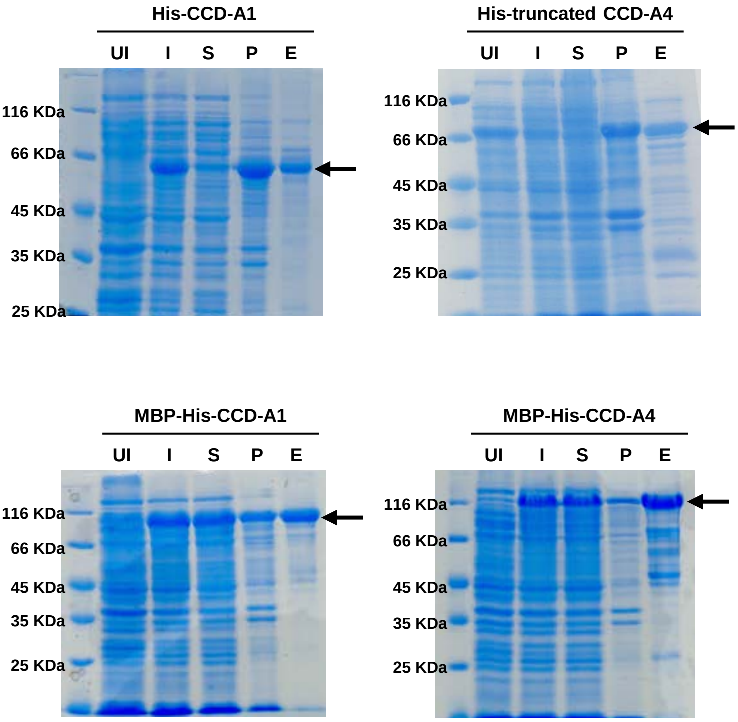

Figure S2. *E. coli* expression and purification of wheat CCD1 and CCD4 proteins. Representative induction and purification of His-tagged CCD-A1, His-tagged truncated CCD-A4, as well as MBP-His-tagged CCD-A1 and CCD-A4 are shown. UI, uninduced; I, induced; S, supernatant; P, pellet; E, eluted purified proteins.
