# Supplementary material for: Distinct expression and function of carotenoid metabolic genes and homoeologs in developing wheat grains
Source: BMC Plant Biol. 2016 Jul 12;16:155. doi: 10.1186/s12870-016-0848-7 (PMC4943016; doi:10.1186/s12870-016-0848-7)
Supplement: Additional file 6: Figure S4. — In vitro activities of wheat CCD-D1 and CCD-D4 homoeologs. (PDF 148 kb) [file 12870_2016_848_MOESM6_ESM.pdf]

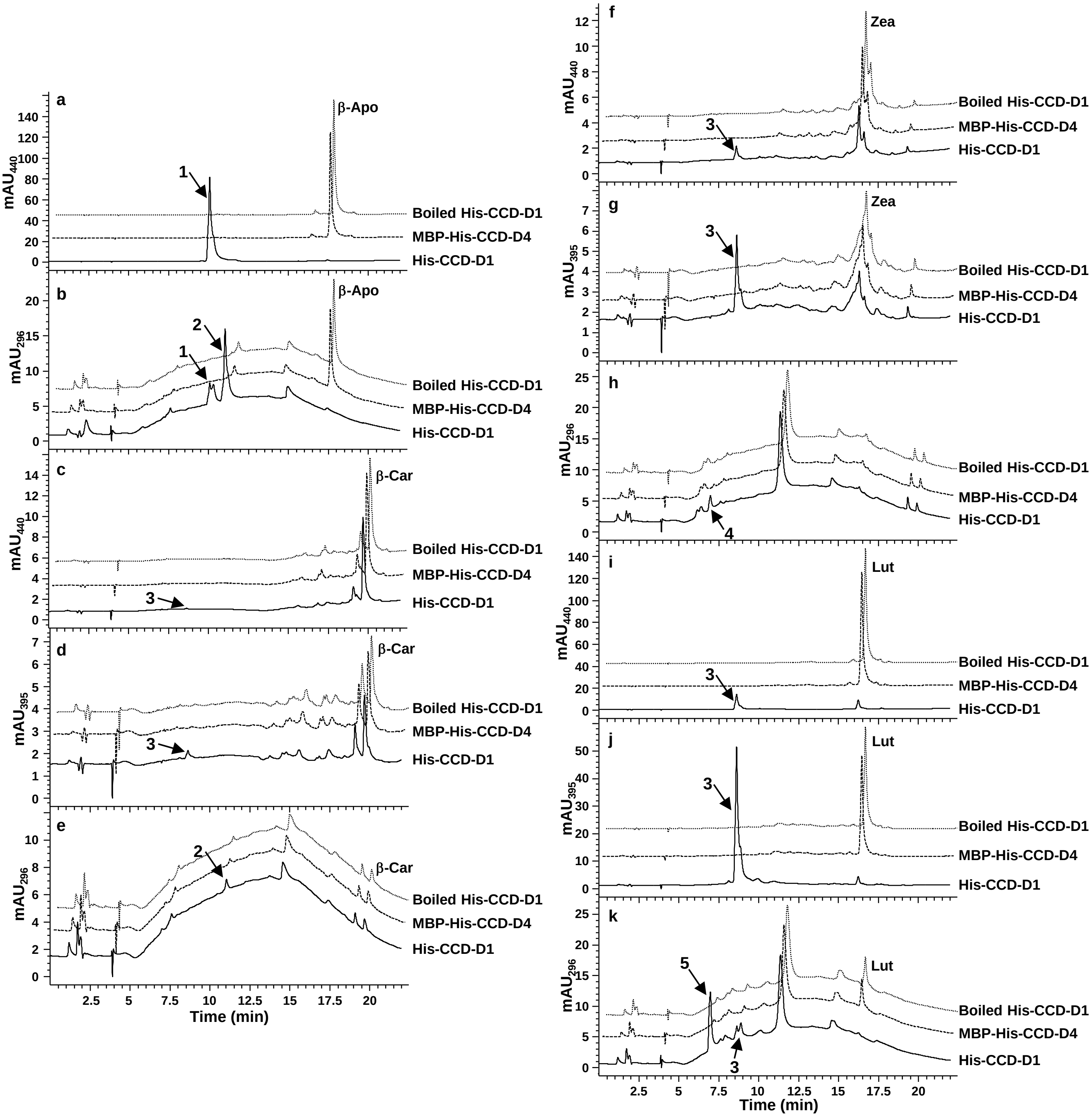

Figure S4. *In vitro* activities of wheat CCD-D1 and CCD-D4 homeologs. β-apo-8'-carotenal (β-apo; a,b), β-carotene (β-car; c-e), zeaxanthin (Zea; f-h), and lutein (Lut; i-k) were used as substrates in the enzyme assays. Product peaks are indicated by arrows. Absorption and MS spectra of the product peaks are shown in Fig. 3.
