# Supplementary material for: Distinct expression and function of carotenoid metabolic genes and homoeologs in developing wheat grains
Source: BMC Plant Biol. 2016 Jul 12;16:155. doi: 10.1186/s12870-016-0848-7 (PMC4943016; doi:10.1186/s12870-016-0848-7)
Supplement: Additional file 7: Figure S5. — Characterization of wheat CCD-B1, CCD-B4, CCD-D1 and CCD-D4 activities in β-carotene producing E. coli cells. (PDF 45 kb) [file 12870_2016_848_MOESM7_ESM.pdf]

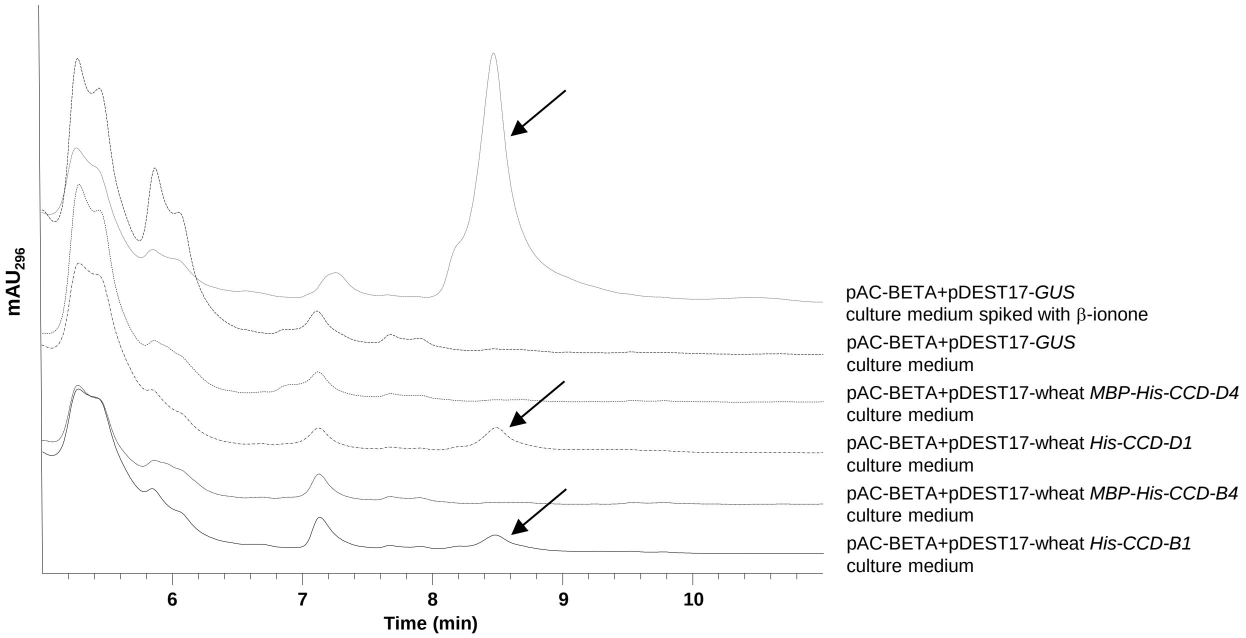

Figure S5. Characterization of wheat CCD-B1, CCD-B4, CCD-D1 and CCD-D4 activities in β-carotene producing *E. coli* cells. pAC-BETA contains all the genes for β-carotene biosynthesis. Total carotenoids extracted from growth media of *E. coli* cells co-expressing pAC-BETA and various plasmid constructs were analyzed by HPLC. The HPLC elution profiles between 5 and 11 min are shown. Ascending HPLC traces are offset by 12 s for clarity. β-ionone is indicated by arrow.
