# Supplementary material for: Distinct expression and function of carotenoid metabolic genes and homoeologs in developing wheat grains
Source: BMC Plant Biol. 2016 Jul 12;16:155. doi: 10.1186/s12870-016-0848-7 (PMC4943016; doi:10.1186/s12870-016-0848-7)
Supplement: Additional file 8: Figure S6. — In vitro activities of truncated wheat CCD4 homoeologs. (PDF 259 kb) [file 12870_2016_848_MOESM8_ESM.pdf]

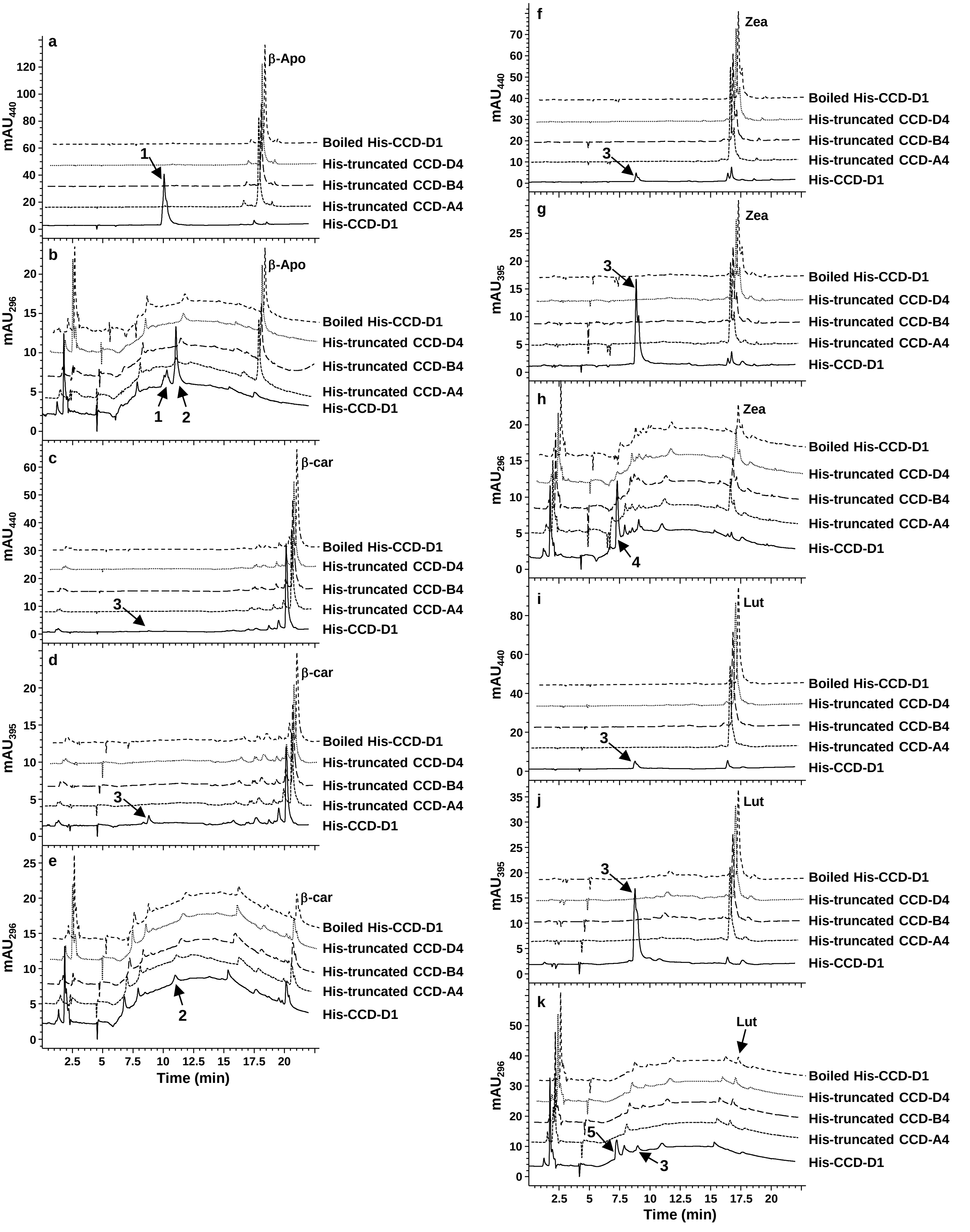

Figure S6. ***In vitro*** **activities of truncated wheat CCD4 homeologs.** β-apo-8'-carotenal (β-apo; a,b), β-carotene (β-car; c-e), zeaxanthin (Zea; f-h), and lutein (Lut; i-k) were used as substrates in the enzyme assays. Product peaks are indicated by arrows. Absorption and MS spectra of the product peaks are shown in Fig. 3.
